# Chest compression quality and patient outcomes with the use of a CPR feedback device: A retrospective study

**DOI:** 10.1038/s41598-023-46862-x

**Published:** 2023-11-13

**Authors:** Wen Zhe Leo, Damien Chua, Hui Cheng Tan, Vui Kian Ho

**Affiliations:** 1 Lee Kong Chian School of Medicine, 11 Mandalay Road, Singapore, 308232 Singapore; 2 Department of Clinical Governance, Sengkang General Hospital, 110 Sengkang East Way, Singapore, 544886 Singapore; 3 Department of Intensive Care Medicine, Sengkang General Hospital, 110 Sengkang East Way, Singapore, 544886 Singapore

**Keywords:** Cardiology, Arrhythmias

## Abstract

Feedback devices were developed to guide resuscitations as targets recommended by various guidelines are difficult to achieve. Yet, there is limited evidence to support their use for in-hospital cardiac arrests (IHCA), and they did not correlate with patient outcomes. Therefore, this study has investigated the compression quality and patient outcomes in IHCA with the use of a feedback device via a retrospective study of inpatient code blue activations in a Singapore hospital over one year. The primary outcome was compression quality and secondary outcomes were survival, downtime and neurological status. 64 of 110 (58.2%) cases were included. Most resuscitations (71.9%) met the recommended chest compression fraction (CCF, defined as the proportion of time spent on compressions during resuscitation) despite overall quality being suboptimal. Greater survival to discharge and better neurological status in resuscitated patients respectively correlated with higher median CCF (*p* = 0.040 and 0.026 respectively) and shorter downtime (*p* < 0.001 and 0.001 respectively); independently, a higher CCF correlated with a shorter downtime (*p* = 0.014). Overall, this study demonstrated that reducing interruptions is crucial for good outcomes in IHCA. However, compression quality remained suboptimal despite feedback device implementation, possibly requiring further simulation training and coaching. Future multicentre studies incorporating these measures should be explored.

## Introduction

The provision of high-quality cardiopulmonary resuscitation (CPR) is a fundamental skill which has significant impact on patient’s survival and prognosis^1–4^. The 5 pillars of high-quality CPR as described by the American Heart Association (AHA) in 2015 are chest compression fraction (CCF) of above 80%, compression rate between 100 to 120 per minute, compression depth greater or equal to 5 cm but not exceeding 6 cm in an adult, no residual leaning and no excessive ventilation^5^. However, CPR providers often do not meet such standards^6–8^. Therefore, to help providers achieve such targets, the AHA stated that it was “reasonable” for CPR providers to utilise a feedback device during resuscitation^3^. This is especially important as deaths from cardiovascular disease, including sudden cardiac death, are the leading cause of mortality worldwide^9,10^.

Out-of-hospital cardiac arrests (OHCA) alone contributed to over 60% of cardiovascular deaths globally in 2010^10,11^. Hence, many papers have reviewed the effectiveness of these feedback devices in the setting of OHCA. Other papers reviewed CPR quality when compressions were performed on mannikins in a controlled and simulated environment^12–14^. Based on these studies, some have recommended the use of automated feedback to improve CPR quality^15,16^.

However, studies on IHCA have been fairly limited despite these patients often having worse outcomes^1,4,17^. This could be contributed by the poorer baseline condition due to multiple comorbidities^18^. Altogether, few studies have been conducted on CPR feedback devices used in IHCA but they were unable to capture the full spectrum of compression parameters including CCF. It was also difficult to draw meaningful conclusions from these studies as results were often indeterminate^19–21,23^. These studies were also unable to establish a relationship between the provision of high-quality CPR with a feedback device and patient outcomes^12,15,16,21^. Hence, this study aims to examine CPR quality with the use of a feedback device during actual code blue activations and if the CPR quality correlates with outcomes in IHCA patients.

## Methods

### Study design

The waiver for this retrospective study was granted by the SingHealth Centralised Institutional Review Board (IRB) (Reference Number: 2019/2496). The methods employed in this study were in accordance to the relevant guidelines and regulations of the IRB. This manuscript was written in accordance to the STROBE checklist on cross-sectional studies.

All inpatient code blue activations from June 2020 to May 2021 in Sengkang General Hospital (SKH) was conducted using inpatient medical records. The inpatient resuscitation trolleys were equipped with the ZOLL® R Series® Plus defibrillator with ZOLL® Stat-padz® (ZOLL Medical Corporation, Chelmsford, MA, United States of America). The feedback device provided instantaneous audiovisual feedback via the International R Series® Software. Compression parameters from the feedback device were then uploaded via wifi for storage and then retrieved using the manufacturer’s software (RescueNet® CaseReview). Patients were subsequently reviewed for their survival and outcomes.

A prior study had been conducted by the ZOLL Medical Corporation to determine the accuracy of the feedback system^24^. In this study, 20 laypersons performed 45 to 60 s of continuous chest compressions on an instrumented manikin. Both the feedback device and the manikin recorded compression rate and depth independently. The data from the feedback device and manikin were analysed and compared against each other. Compared to the data from the manikin, the compression depth measured by the feedback device varied by a mean of 0.13 ± 0.64 cm (95% confidence interval) and the compression rate differed by an average of less than 2 compressions per minute.

### Resuscitation model in Singapore

Code blue teams (CBT) in Singapore are activated for arrest and peri-arrest cases. Peri-arrest cases involve patients who are unstable and very likely go into cardiac arrest such as impending airway collapse and refractory shock. Upon cardiac arrest, the junior doctor of the ward team is notified and activated to attend to the patient. He or she attends swiftly to the collapsed patient and leads the basic resuscitation effort with the on-scene nursing staff and further activates the CBT at the soonest opportunity. The CBT then takes over the resuscitation effort from the ward team upon arrival. Most, if not all, patients who attain return of spontaneous circulation (ROSC) will be transferred to the Intensive Care Unit (ICU) for further management by the ICU team.

### Patient population

Waiver for informed consent was granted by the SingHealth Centralised IRB (Reference Number: 2019/2496). All patients in SKH and Sengkang Community Hospital (SKCH) who had a cardiopulmonary collapse in areas covered by the inpatient CBT were included. These areas included the general ward, high dependency unit, endoscopy suite, dialysis centre, cardiovascular laboratory and SKCH inpatient wards.

Patients excluded were those who: (1) had achieved ROSC upon the CBT arrival; or (2) were on a Do Not Resuscitate (DNR) order. Peri-arrest activations which ultimately did not go into cardiac arrest were excluded from the analysis.

### Measured outcomes

Primary outcome variables were CCF, proportion of compressions in target, mean compression rate and proportion of compressions in target rate, mean compression depth and proportion of compressions in target depth, and mean release velocity (RV). CCF was defined as proportion of time during resuscitation that chest compressions were performed. Compressions in target were defined as compressions that met both target rate and depth concurrently. RV was used as a surrogate marker for chest recoil. The compression targets were assessed using recommendations by AHA^5^.

Secondary outcome variables were downtime, survival and neurological outcomes. Downtime was defined as the interval from cardiopulmonary collapse to sustained ROSC, in which spontaneous circulation had been achieved without the need for further chest compressions for at least 20 min^25^. Survival of patients was recorded at hospital discharge.

A patient’s neurological function was assessed using the Cerebral Performance Categories (CPC). The CPC is a commonly used modality to prognosticate long-term neurological function after a cardiac arrest. The categories are: (1) good cerebral performance; (2) moderate cerebral disability; (3) severe cerebral disability; (4) coma, vegetative state; and (5) dead^26^.

### Statistical analysis

Data was recorded using Microsoft® Excel® 365 software (Microsoft Corporation, Redmond, WA, United States of America). Missing data were reconciled where possible. Data which were unable to be reconciled were excluded from the analysis. Analysis was performed using IBM SPSS Statistics 28 software (IBM Corporation, Armonk, NY, United States of America). Shapiro–Wilk test was used to determine if continuous variables were normally distributed.

Due to the small sample size within subgroups, continuous variables were presented as median with interquartile range (IQR). Categorical variables were presented as numbers or percentages (%).

Mann–Whitney U and Kruskal–Wallis tests were performed to determine if there were significant differences between 2 or more than 2 outcome categories measured on a continuous scale. Measurements for compression parameters and downtime were collected. The data were analysed using the Kruskal–Wallis test to detect significant differences in medians of parameters and downtime for all survival outcome categories. Post-hoc tests were performed where necessary using Bonferroni correction, adjusting for multiple pairwise comparisons. Results were taken to be statistically significant if *p* < 0.05.

## Results

### Patient demographics

The data was collected from June 2020 to May 2021. Amongst the 110 code blue activations, 34 cases (30.9%) had no data captured and 5 cases (4.5%) had more than or equal to a 10% discrepancy in the number of data points captured for depth and rate. The exclusion criteria omitted 7 cases, of which, 2 cases (1.8%) had an active DNR order while 5 cases (4.5%) achieved ROSC upon CBT arrival. Altogether, 64 cases were ultimately included for analysis.

Overall, there were more males than females. The majority of patients were Chinese elderly (mean age: 71.4 ± 12.2 years). The most common organ system causing initial collapse was the cardiovascular system. Most patients had non-shockable rhythms (pulseless electrical activity or asystole) (74%) as their initial rhythms and 67% of patients did not receive any shock throughout resuscitation. The 10 discharged patients mostly returned home or were transferred to a stepdown care facility (Table 1).

**Table 1:** Characteristics of patients included in this study. SD: Standard Deviation; IQR: Interquartile Range.

	Overall (n = 64)
Age (in years)	
Mean (SD)	71.4 (12.2)
Median (IQR)	72.5 (61.3–78.8)
Gender, n (%)	
Female	25 (39.1)
Male	39 (60.9)
Ethnicity, n (%)	
Chinese	48 (75.0)
Malay	11 (17.2)
Indian	5 (7.8)
Organ system causing initial collapse, n (%)	
Cardiovascular	25 (39.1)
Respiratory	15 (23.4)
Metabolic/ renal	5 (7.8)
Gastrointestinal	1 (1.6)
Neurological	1 (1.6)
Others	17 (26.6)
Initial rhythms, n (%)	
Pulseless electrical activity	24 (37.5)
Asystole	23 (35.9)
Ventricular fibrillation	7 (10.9)
Normal sinus rhythm	3 (4.7)
Sinus tachycardia	1 (1.6)
Ventricular tachycardia	1 (1.6)
Unknown	5 (7.8)
Discharge destination, n (%)	(n = 10)
Home	4 (40.0)
Stepdown care facility	4 (40.0)
Nursing home	1 (10.0)
Other acute wards	1 (10.0)

The median response time from CBT activation to CBT arrival on the scene was 4.0 (3.0–5.0) minutes. The rates of ROSC and survival to hospital discharge were 64.1% and 13.7% respectively.

### Compression quality was suboptimal

Compression parameters were retrieved from the manufacturer’s software. CPR performances were subsequently assessed using AHA recommendations.

71.9% of cases achieved CCF of above 80%. While 56.3% and 39.1% of compressions were within the target ranges of rate and depth respectively, only 13.7% (7.1–24.5%) of compressions met the composite of the two. No compression met the target RV.

### Resuscitation outcome was not associated with any one compression parameter

With regards to initial survival outcome, we explored possible associations between compression parameters and outcome of resuscitation. Survival outcome from CPR was divided into those who: (1) did not attain ROSC; and (2) attained ROSC. No statistically significant difference was observed between CCF, depth, rate and RV for CPR survival outcome (*p* = 0.40, 0.93, 0.94 and 0.82 respectively) (Table 2).

**Table 2:** Associations between the various chest compression parameters and CPR survival outcomes.

	CPR survival outcome			
	Did not attain ROSC (n = 25)	Attained ROSC (n = 39)	Overall (n = 64)	*p*-value^1^
Chest compression fraction (in %)				
Median (IQR)	84.7 (74.7–90.9)	87.6 (77.2–93.1)	86.6 (77.0–92.3)	0.401
Chest compression depth (in cm)				
Median (IQR)	5.4 (5.0–5.8)	5.5 (4.95–5.88)	5.44 (5.02–5.84)	0.934
Chest compression rate (per minute)				
Median (IQR)	121.9 (112.6–126.3)	120.5 (113.5–126.6)	120.8 (113.3–126.4)	0.940
Release velocity (in cm/s)				
Median (IQR)	25.6 (23.3–27.5)	25.6 (23.3–28.4)	25.6 (23.8–28.0)	0.820

CPR: Cardiopulmonary Resuscitation; ROSC: Return of Spontaneous Circulation; IQR: Interquartile Range.

^1^Mann-Whitney U test.

### Patient prognosis after ROSC correlated with CCF and downtime

Next, we investigated if resuscitation parameters were associated with patient survival. The patients who attained ROSC were categorised into those who: (1) did not survive till hospital discharge; and (2) survived till hospital discharge.

The overall median CCF and downtime for patients who attained ROSC were 86.6% (77.0–92.3%) and 15.0 (7.5–20.3) minutes respectively. Amongst these patients who attained ROSC, the median CCF was higher in those who “survived till hospital discharge” (92.3%) than those who “did not survive till hospital discharge” (86.8%). Further, the median downtime was also shorter in the former, by a difference of 11.5 min. These differences in CCF and downtime, in terms of patients who survival till hospital discharge or not, were statistically different (*p* = 0.04 and < 0.001 respectively). However, no statistically significant difference was found for depth, rate and RV for these survival categories (*p* = 0.69, 0.96 and 0.79 respectively) (Table 3).

**Table 3:** Associations between the various chest compression parameters and downtime, and survival till hospital discharge after ROSC.

	Ultimate survival outcome			
	Did not survive till hospital discharge (n = 29)	Survived till hospital discharge (n = 10)	Overall (n = 39)	*p*-value^1^
Chest compression fraction (in %)				
Median (IQR)	86.8 (76.3–90.7)	92.3 (84.5–98.7)	86.6 (77.0–92.3)	**0.040**
Chest compression depth (in cm)				
Median (IQR)	5.5 (5.2–5.8)	5.2 (4.9–6.1)	5.4 (5.0–5.8)	0.692
Chest compression rate (per minute)				
Median (IQR)	120.8 (113.4–127.3)	119.9 (113.1–131.1)	120.8 (113.3–126.4)	0.962
Release velocity (in cm/s)				
Median (IQR)	26.3 (23.5–28.2)	24.7 (22.1–29.5)	25.6 (23.8–28.0)	0.788
Downtime (min)	(n = 28)	(n = 10)	(n = 38)	
Median (IQR)	17.5 (10.5–22.0)	6.0 (2.0–10.5)	15.0 (7.5–20.3)	** < 0.001**

ROSC: Return of Spontaneous Circulation; IQR: Interquartile Range.

^1^Mann-Whitney U test.

Significant values are in bold.

### CPC correlated with CCF and downtime respectively

To investigate the extent in which neurological status is affected after resuscitation, we explored how the compression parameters correlated with CPC. We observed statistically significant differences in median CCF and downtime with CPC at discharge (*p* = 0.03 and 0.001 respectively). Median CCF (92.3%) was higher in patients with CPC = 1 than in patients with CPC = 5 (86.4%). Moreover, median downtime (6.0 min) was also shorter in patients with CPC = 1 than in patients with CPC = 5 (17.5 min). However, no statistically significant difference was observed in compression depth, rate and RV with CPC at discharge (*p* = 0.68, 0.97 and 0.77 respectively) (Table 4).

**Table 4:** Associations between the various chest compression parameters and downtime, and CPC.

	Cerebral performance categories			
	CPC = 1 (n = 10)	CPC = 5 (n = 54)	Overall (n = 64)	*p*-value^1^
Chest compression fraction (in %)				
Median (IQR)	92.3 (84.5–98.7)	86.4 (76.4–90.7)	86.6 (77.0–92.3)	**0.026**
Chest compression depth (in cm)				
Median (IQR)	5.2 (4.9–6.1)	5.5 (5.1–5.8)	5.4 (5.0–5.8)	0.677
Chest compression rate (per minute)				
Median (IQR)	119.9 (113.1–131.1)	121.4 (113.2–126.1)	120.8 (113.3–126.4)	0.971
Release velocity (in cm/s)				
Median (IQR)	24.4 (22.7–26.1)	25.6 (23.5–28.4)	25.6 (23.5–28.4)	0.767
Downtime (min)	(n = 10)	(n = 28)	(n = 38)	
Median (IQR)	6.0 (2.0–10.5)	17.5 (10.5–22.0)	15.0 (7.5–20.3)	**0.001**

CPC: Cerebral Performance Categories; IQR: Interquartile Range.

^1^Mann-Whitney U test.

Significant values are in bold.

### CCF independently correlated with downtime

This study also examined whether compression parameters correlated with downtime. Downtimes were categorised into: (1) short (< 10 min); (2) moderate (10–20 min); and (3) long (> 20 min). We observed that a longer duration of downtime was associated with lower CCF (p = 0.014). Median CCF for: (1) short downtime was 92.3%; (2) moderate downtime was 87.0%; and (3) long downtime was 74.0%. Furthermore, post-hoc Bonferroni correction showed a statistically significantly higher CCF for those with short downtime than those with long downtime (*p* = 0.012) (Table 5).

**Table 5:** Associations between the various chest compression parameters and downtime for patients who attained ROSC.

	Downtime				
	Short (n = 14)	Moderate (n = 15)	Long (n = 9)	Overall (n = 38)	*p*-value^1^
Chest compression fraction (in %)					
Median (IQR)	92.3 (84.7–98.4)	87.0 (77.2–90.6)	74.0 (58.1–88.4)	86.6 (77.0–92.3)	**0.014**^**2**^
Chest compression depth (in cm)					
Median (IQR)	5.4 (4.9–6.1)	5.6 (5.2–6.1)	5.5 (5.2–5.8)	5.4 (5.0–5.8)	0.877
Chest compression rate (per minute)					
Median (IQR)	122.1 (113.1–134.9)	119.6 (113.5–125.2)	119.4 (111.5–124.1)	120.8 (113.3–126.4)	0.510
Release velocity (in cm/s)					
Median (IQR)	25.8 (22.1–28.9)	25.6 (23.3–29.2)	26.3 (23.9–30.1)	25.6 (23.8–28.0)	0.904

ROSC: Return of Spontaneous Circulation; IQR: Interquartile Range.

^**1**^Kruskal-Wallis test.

^2^Bonferroni correction was performed for multiple tests between groups. There was statistically significant difference in CCF for those who had a short downtime than those who had a long downtime (*p* = 0.012).

Significant value is in bold.

## Discussion

This study examined CPR quality with a feedback device and how CPR performance correlated with outcomes of IHCA patients. Herein, under the guidance of the CPR feedback device, we identified that increased CCF and shorter downtime were associated with improved CPR survivability and neurological prognosis of patients. This study provided nascent insights and thorough evaluation on the execution of CPR. It also explored the potential avenues in resuscitation medicine in which CPR training could be further modified to potentially improve patient outcomes.

### Compressions required further guidance

Abella et al. reported in 2005 that CPR providers averaged a mean compression rate of 105 per minute while Wiks et al. reported a mean compression rate of 121 per minute which improved to 109 compressions per minute with feedback^6–8^. Similarly, the former reported an average depth of 4.3 cm while the latter observed an average depth of 3.4 cm, which improved to 3.8 cm with feedback^6–8^. Compression depth in target also improved from 28 to 53% with feedback, as demonstrated by studies in 2005 and 2006 respectively^7,8^. Overall, it was observed that despite the use of a similar feedback device in 2006, compressions remained inadequate even for the guidelines at that time^6–8^.

Kovacs et al. found that an RV equal to or exceeding 40 cm/s was associated with improved survival and neurological outcomes^27^. Based on the abovementioned study and AHA 2015 guidelines, the CPR quality in this study was suboptimal. There was inadequate chest recoil and the median compression rate was also above target^5^. Overall, there was a low percentage of compressions in target. These occurred despite real-time feedback. Achieving full chest recoil is difficult^28^. High levels of self-reported stress were also reported to be associated with decreased CPR performance^29^. Even with the implementation of the feedback device, quality was still inadequate and calls for further intervention to improve CPR quality.

Thus, one solution is for CBT to introduce the role of a CPR coach. The CPR coach deduces compression quality based on the feedback generated from the CPR feedback device and first-hand observation to guide the provider in making adjustments to achieve the CPR targets. The CPR coach may be the CBT leader or assigned to another CBT member. Over the years, some had attempted to review the effectiveness of CPR coaching in resuscitation. While these studies were mostly limited to OHCA and paediatric IHCA, they demonstrated that CPR coaching improved adherence to resuscitation guidelines^30–33^. Most pertinently, a 2022 South Korean study which looked into a novel OHCA resuscitation protocol (which included CPR coaching) showed substantial improvements in the rates of prehospital ROSC, survival till hospital discharge and favourable neurological status^34^. It is crucial that future reviews of CPR data explore whether the introduction of a CPR coach, especially in adult resuscitation with prolonged downtime, leads to improved compression parameters and if such improvements correlate with better survival and neurological outcomes.

Moreover, with the variability of CPR quality between providers, more training to standardise quality is likely necessary. Simulation training was reported to be most effective in training to improve CPR quality^16,35^. Therefore, training sessions should also include the CPR coach in simulations to enable the CBT to incorporate this role while mimicking stressful real-life conditions.

### Minimising interruptions during CPR is essential for good outcomes in IHCA patients

This study demonstrated that a higher CCF was associated with shorter downtime. Current evidence corroborates with our findings where a higher CCF reduces mortality and morbidity in patients who suffered from a cardiac arrest^36–38^. Additionally, a shorter downtime was also associated with better survival to hospital discharge and neurological status, supporting our findings^39,40^.

**Figure 1:**
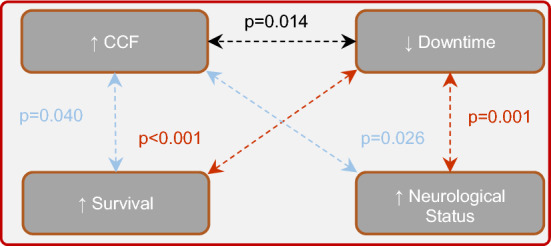
Associations between CCF and downtime, and patient survival and neurological status | Increased survival and better neurological status of patients were individually associated with a higher CCF (*p* = 0.040 and 0.026 respectively) and a shorter downtime (*p* < 0.001 and 0.001 respectively). A shorter downtime was independently associated with a higher CCF (*p* = 0.014).

It is noteworthy that the current AHA recommendation to minimise interruptions during CPR are based on papers which studied OHCA patients^5,41^. Past studies also did not find reduced interruptions to be associated with improved survival and neurological status in IHCA patients^38^. Hence, it is reasonable to postulate that achieving uninterrupted compressions is important for good outcomes in IHCA patients, and this further strengthens AHA recommendation to minimise interruptions during CPR for OHCA and now IHCA patients (Fig. 1).

With a median CCF of above 80%, this study has demonstrated that such a target is achievable within the hospital setting when performing resuscitations on IHCA patients. This supports current expert consensus^41^. This is an important finding since high CCF is associated with better patient outcomes.

However, it is currently unclear whether CCF decreases over time with prolonged CPR due to fatigue, or whether having a high CCF directly reduces downtime. Therefore, future studies can further explore this association.

### Other confounders

The use of analysis of means difference does not exclude other confounders from contributing to the differences. Therefore, spurious correlations may be generated. For example, the quality of post-resuscitation care may be different among various patients, which is an important factor that affects the prognosis of patients^42^. It is thus included as one of the steps in the chain of survival for all cardiac arrest patients^43,44^. Moreover, non-modifiable variables such as the patient’s gender, age and prior comorbidities affect outcomes, but cannot be reliably excluded for comparison and discussion^45^.

### Limitations

Firstly, the sample size was small (n = 64). As such, this study is potentially underpowered to conclude the effects of compression parameters. A future multicentre study can increase the sample size. Secondly, there was significant data loss (n = 34; 31%). Data loss over wifi is being rectified for future evaluations. Thirdly, this study was unable to verify self-reported variables like patient downtime and CPC. Such variables were entered as a free-text note. Unlike retrieving compression parameters via the manufacturer’s software, a retrospective accuracy check for free-text notes was not possible. Lastly, this study was unable to capture pauses during CPR (for example, pulse check, perishock and intubation) and time from collapse to CPR. These affect survival and patient outcomes^3,46–48^. The data collection methodology will be revised to include these in future studies. This study was ultimately a retrospective one. It cannot control for all confounders or support causal relationships^49^.

### Areas for further research

Only a few studies utilising a feedback device have been conducted on humans^6,8,19–21,50^. Few were conducted on IHCA^6,19–21^. This is perhaps the first study to review the effectiveness of a CPR feedback device which provided depth, rate and RV feedback during IHCA. At the time of writing, there are ongoing studies using real-time feedback on these parameters conducted in Japan and England^51,52^.

## Conclusions

CPR quality was suboptimal despite using a CPR feedback device to guide resuscitation. CPR providers and the CBT included in this study require further training with on-site CPR coaching. Despite its limitations, this study revealed that achieving higher CCF is achievable and essential for good outcomes in IHCA patients who achieve ROSC, further strengthening AHA recommendation to minimise interruptions during CPR. Nonetheless, future multicentre studies with a greater sample size exploring the clinical effects of CPR feedback device implementation will be helpful.

However, it should be emphasised that providing high-quality CPR is merely one factor which impacts the outcomes of IHCA patients. Patient outcomes are also dependent on a robust system involving training, protocols and post-resuscitation care^16,35,43,44^. Therefore, optimisation of post-resuscitation care and review of current systems are also essential in improving the prognosis of cardiac arrest patients^43,44,53^.

## Data Availability

All data generated or analysed during this study are included in this published article.
